# Remote Cell Growth Sensing Using Self-Sustained Bio-Oscillations

**DOI:** 10.3390/s18082550

**Published:** 2018-08-03

**Authors:** Pablo Pérez, Gloria Huertas, Alberto Olmo, Andrés Maldonado-Jacobi, Juan A. Serrano, María E. Martín, Paula Daza, Alberto Yúfera

**Affiliations:** 1 Instituto de Microelectrónica de Sevilla, IMSE, CNM (Universidad de Sevilla, CSIC), Av. Américo Vespucio, SN, 41092 Sevilla, Spain; aolmo@dte.us.es (A.O.); maldonado@imse-cnm.csic.es (A.M.-J.); serrano@imse-cnm.csic.es (J.A.S.); yufera@imse-cnm.csic.es (A.Y.); 2 Departamento de Tecnología Electrónica, Escuela Técnica Superior de Ingeniería Informática, Universidad de Sevilla, Av. Reina Mercedes, SN, 41012 Sevilla, Spain; 3 Departamento de Electrónica y Electromagnetismo, Facultad de Física, Universidad de Sevilla, Av. Reina Mercedes, SN, 41012 Sevilla, Spain; 4 Departamento de Biología Celular, Facultad de Biología, Universidad de Sevilla, Av. Reina Mercedes, SN, 41012 Sevilla, Spain; mariamartin@us.es (M.E.M.); pdaza@us.es (P.D.)

**Keywords:** smart sensing, bioimpedance, cell culture, OBT, real-time monitoring

## Abstract

A smart sensor system for cell culture real-time supervision is proposed, allowing for a significant reduction in human effort applied to this type of assay. The approach converts the cell culture under test into a suitable “biological” oscillator. The system enables the remote acquisition and management of the “biological” oscillation signals through a secure web interface. The indirectly observed biological properties are cell growth and cell number, which are straightforwardly related to the measured bio-oscillation signal parameters, i.e., frequency and amplitude. The sensor extracts the information without complex circuitry for acquisition and measurement, taking advantage of the microcontroller features. A discrete prototype for sensing and remote monitoring is presented along with the experimental results obtained from the performed measurements, achieving the expected performance and outcomes.

## 1. Introduction

Biological cell culture assays performed in biomedical research laboratories are of vital importance [1]. However, estimating the cell growth on test plates is time-consuming and requires intensive human supervision during the whole process, introducing a large variability factor to assays. Characterizing the number of cells in a culture at a specific time, as well as measuring the cell proliferation rate, have deep implications in biomedicine, both at the technical and biological levels. The cell density in the plate will indicate the success of many different techniques, including transfection, infection, chemical response, etc. Cells distributed too sparsely will have problems growing and will react strangely to all the treatments. More important from a biological point of view, cell proliferation is a significant figure of merit to characterize a cell line. Excessive proliferation is the hallmark of cancer cells, and any physical or chemical treatments slowing down cell growth may lead to potential oncological therapies. Lack of proliferation usually represents excessive cell death, either due to extrinsic or intrinsic mechanisms, or the activation of cellular aging pathways (cellular senescence). Thus, understanding the processes that slow down or accelerate the proliferation of a culture, including the dynamics of these processes, is a powerful research tool [1,2].

However, despite its importance, biologists face serious difficulties in monitoring cell proliferation in detail. There are two key technical problems. First, protocols to estimate the number of cells in a plate are heavily invasive, and profoundly affect the biology of the cells. In most cases, once a measurement is carried out, the involved cells can no longer be used for subsequent experiments (end-point assays). Secondly, scientists can only take snapshots of the culture at specific instances. Thus, for most biomedical researchers, the only alternative is to set up many parallel experiments and reconstruct the progression of a culture using all these experiments in an aggregate way. Furthermore, in many cases this will be a retrospective analysis, and understanding how cells have behaved can only be done at the end of the experiment with low insight into its dynamics. For these reasons, any new technical approach facilitating cell progression monitoring (especially if real-time on-line monitoring is provided) will have an immediate impact in biological practice. It is worthwhile to develop measurement techniques with a simple set up that would allow researchers to monitor the evolution of their experiments on-line without perturbing the involved cells.

This work aims to provide a procedure to support living cell assays with a simple on-line and remote non-invasive measurement capabilities in such a way that an operator (a biomedical researcher) can remotely and in real-time pursue the evolution of cell cultures through a web tool displaying the acquired data. In the article, a system for performing this supervisory task is proposed using inexpensive discrete components. The global system is implemented using an oscillation-based test (OBT) sensor which is widely described in [3,4,5], based on an ARM Cortex-M7 microcontroller and an Intel Edison device as the external system-on-chip which communicates with the sensor using a wireless link based on Bluetooth technology. The peripheral blocks which provide the system with smart features are defined in detail herein.

The OBT approach includes the “biological” sample under test in the path of the oscillation loop, hence forming a bio-oscillator, scanning and encoding the valuable biological information on the signal being generated by the circuit [3,4,5]. A detailed model of the used electrode allows us to include biological properties [5,6,7,8]. The oscillation parameters such as amplitude, frequencies of the main harmonics, phase, etc., are directly related to the biological variables under study, such as cell number, cell growth rate, cell size, etc., linking the biological properties to the electrical ones [3,4,5]. The biological interface connected to the proposed self-sustained nonlinear feedback loop, the bio-oscillator, is made of a set of microelectrodes and the cells immersed in a cell culture medium. This interface requires precise modeling to obtain an accurate estimation of cell occupation and cell growth in the biological sample under test [5,9,10,11,12].

In summary, the whole smart sensor proposed in this paper provides remote supervision of cell cultures assays as well as real-time visualization (without interrupting the experiment) of cell occupation and cell growth rate, which are, as was said, of great value for the researchers in a biomedical laboratory. This paper is organized as follows: Section 2 will introduce the smart system architecture and the behavior of each part of this system. Section 3 will present the achieved experimental results from assays with the N2A cell line. Finally, Section 4 and Section 5 will present the discussion and conclusions respectively.

## 2. Smart System Architecture

The system architecture is defined following the paradigms for Internet of Things (IoT) design described in the literature [13]. An IoT architecture presents three main elements: the sensors, the gateway and the services. A sensor is an application-specific device required to meet the necessary accuracy and sampling frequency for the whole process. The gateway is in charge of performing communication tasks between sensors and backend services (data storage and control). It adds the TCP/IP capability, allowing it to be used as a communication link for and between the sensors and services. Finally, the service layer provides the necessary elements to perform secure data storage and/or remote control and visualization.

**Figure 1:**
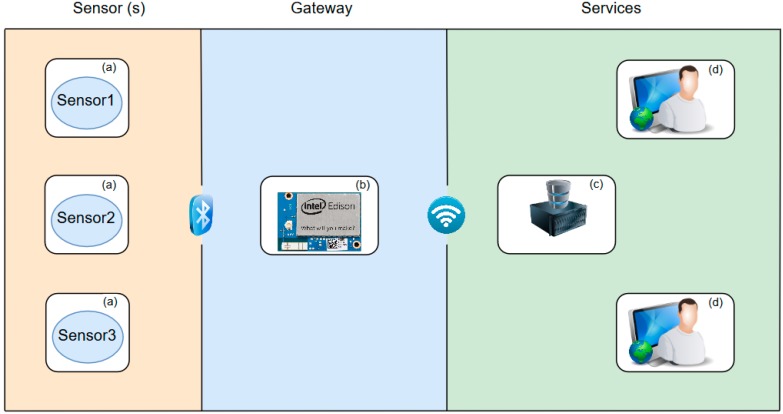
The system architecture. (**a**) represents the sensor devices; (**b**) is the external data acquisition unit based on the Intel Edison gateway; (**c**) is the remote data server which stores data on a MySQL database and provides the secure graphical user interface to the end-users (**d**).

The application described in this paper supports multiple sensors connected at the same time to a unique gateway, which is based on an Intel Edison system-on-chip (SoC). The prototype manages and collects all the information from the available sensor devices, and controls power consumption by setting the devices to low power mode at periodic time lapses. Figure 1 illustrates this architecture. The communication between the gateway and the sensors is performed via Bluetooth.

The sensor devices are custom electronic prototypes that are also capable of measuring humidity, temperature, battery, and the analog sensor electrical properties. As shown above, the sensor devices can also measure the amplitude and frequency of a biological oscillator (OBT approach). The gateway (Intel Edison SoC) manages the experimental measurement protocol according to a set of configurable settings that exist on an online database, provided by a data server through the internet. The final components, the data server and web interface, represent the services layer. These services would be used by the end-user (a biomedical researcher). The user selects measurement rates and experiment start and end times through the secure web interface to begin the experiment and can supervise ongoing experiments through the web interface.

### 2.1. The OBT Sensor

**Figure 2:**
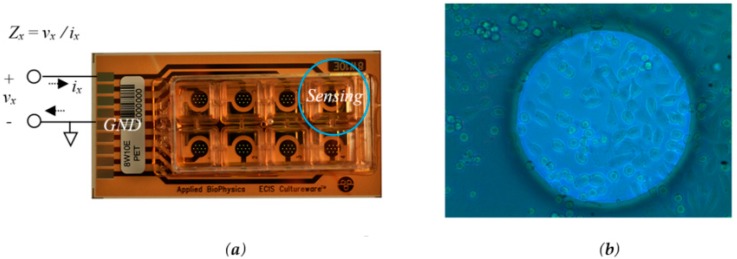
The ECIS Cultureware 8E10W device (**a**); composed of 8 separated wells for cell culture containing a 10 units of 250 µm diameter Au microelectrode. The measure bioimpedance *Z_x_* represents the ratio *v_x_/i_x_*. (**b**) a Chinese hamster ovary fibroblast cell line, AA8 (American Type Culture Collection), cultured over the surface of a single microelectrode.

Our approach uses an electrical cell-substrate impedance spectroscopy (ECIS) sensor [14]. Implementation of an ECIS sensor usually requires complex excitation and acquisition circuits to measure the bioimpedance response of the sample under test. This work takes advantage of this non-invasive impedance-based cell culture technique to reduce both the cost and the human effort. Furthermore, it also decreases the measurement dispersion since it allows a single culture per experimental assay. The electrode solution implemented for this system is provided by Applied Biophysics [15], and is presented on Figure 2.

The biological sample under test is a cell culture formed from mouse neuroblastoma cell lines (the N2A cell line). The cells were cultured in medium consisting of 50% DMEM high glucose (Biowest, Nuaillé, France) and 50% Opti-MEM (Gibco, Alcobendas, Spain) supplemented with 10% (*v*/*v*) foetal bovine serum (FBS) (Gibco), 2 mM *L*-glutamine, 50 μg/mL streptomycin and 50 U/mL penicillin (Sigma-Aldrich, Madrid, Spain). The cells were maintained at 37 °C in a humidified atmosphere with 5% CO_2_ and they were routinely sub-cultured. Different initial numbers of cells were seeded at the well-sensors for our experiments: 2500, 5000 and 10,000. Two well-sensors were filled only with culture medium. Petri plate cultures were also seeded with the same cell densities to further match with the proposed bioimpedance test. The cell count was done over Petri plate preparations for the three initial number of cells: 2500, 5000 and 10,000. The fill factor was estimated considering a N2A cell area value of 184 μm.

#### 2.1.1. Oscillation Based Test Methodology

**Figure 3:**
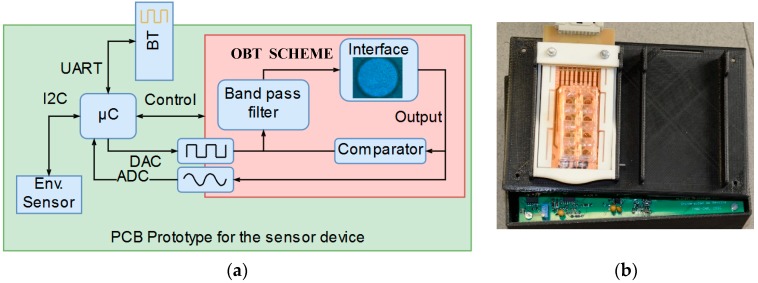
(**a**) The block diagram for the implemented PCB (Printed Circuit Board) prototype of the sensor device. Interdevice communication is performed by data buses; Universal Asynchronous Receiver-Transmitter (UART) and Inter-Integrated Circuit (I2C). The microcontroller (µC) implements Digital to Analog (DAC) and Analog to Digital (ADC) converters to trigger/acquire the Oscillation Based Test (OBT) sensor signal. (**b**) A photo of the sensor device.

**Figure 4:**
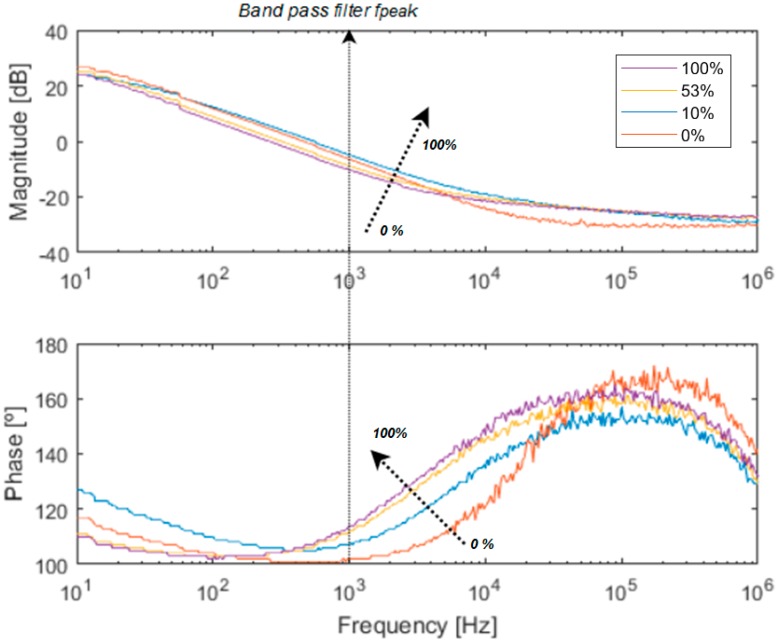
Experimental cell-electrode interface impedance bode (magnitude and phase) for different occupation areas (ff). Each curve corresponds to a different ff in the well from 0 (0%) to 1 (100%). Main pole and zero frequencies are located at 8 Hz and 15 kHz respectively, for ff = 0. The cell line in this experiment is N2A.

The cornerstone of our method is to incorporate the ECIS sensor within the feedback loop of an electronic oscillator, using the OBT methodology depicted in [1,2,3]. The proposed idea is a strategy for the conversion of the cell culture into oscillators, whose characteristic parameters (frequency, amplitude, phase, etc.) are related to the cell culture evolution and can be easily determined. Put simply, a band pass filter followed by the bioimpedance of the cell culture (this is the proposed cell-electrode interface) and a comparator altogether close a nonlinear feedback loop to establish the oscillations (see the inner box on Figure 3, OBT). The closed-loop system is optimized for cell-electrode impedance analysis. Variations on the electrical properties of the cell-electrode complex will generate variations on the oscillation parameters in the oscillation output signal. The cell amount is reflected on an intrinsic parameter of the system which is directly proportional to the number of cells located on the electrodes. This parameter is the fill-factor, which is defined as the percentage of the electrode area covered by cells (always less than one). The fill-factor (ff) or cell-to-electrode overlap area variation generates different values for the impedance being measured by the sensor. Figure 4 displays the typical behavior of an experimental cell-electrode interface bode, showing curves for several cell occupation areas, ff. These curves are directly related to the electrode size, technology and biological material. Thus, to calibrate the bio-oscillator, we must tune the peak frequency (fpeak) of the bandpass filter in the nonlinear feedback loop as indicated in Figure 4. When frequencies around this peak frequency are considered, the system has optimum ff sensitivity for magnitude and phase. This means that both the magnitude and phase response can be correlated to the ff parameter. Most ECIS techniques search for the best frequency response for optimum impedance characterization, and then perform the measurements knowing the ff dependence. Therefore, absolute magnitudes (Figure 4) or normalized magnitudes (CI: cell index) can be used as sensitivity curves for this impedance sensor and assays.

**Figure 5:**
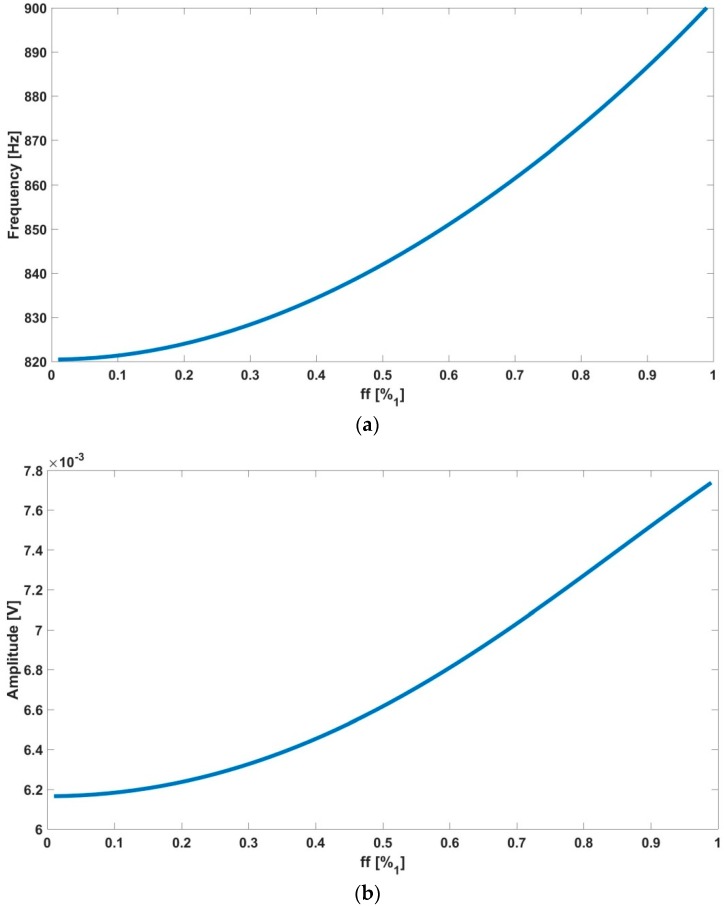
Oscillation parameters, (**a**) frequency and (**b**) amplitude values, obtained with the proposed system using simulations depending on the ff.

On the other hand, the output of the biological oscillator (the input of the nonlinear element, the comparator in Figure 3a) is approximately sinusoidal due to the bandpass characteristics of the global structure. This fact allows us to use the linear approximation stated in the described function method [3,4] for the mathematical treatment of the nonlinear element [16,17]. An oscillatory solution can be found for each fill-factor, ff, which directly correlates the main oscillation parameters with the occupied cell culture area and the number of cells in the culture. Thus, in short, the output signal oscillation parameters, amplitudes and frequencies, are functions of the ff parameter (see [3,4] for mathematical details). Figure 5 illustrates the main harmonic frequency and the amplitude of the predicted oscillation signal for a selected bandpass peak frequency at 1 kHz in our experimental example or study case (Figure 4). For each cell line or type of electrode, the sensitivity of the oscillation parameters regarding the bandpass filter parameters, that is, the peak frequency and the quality factor, has to be determined in order to maximize both the oscillation parameter changes and the dynamic range of the measurements. In Figure 5, the oscillation parameters increase when the cell-to-electrode area overlap (ff) increases from 0 to 1. For each cell line, this behavior will be similar. Thus, acquiring this oscillation output signal is enough to provide accurate measurements of the cell culture status and provide safe and secure data storage for cell dynamics in cell cultures. Moreover, the amount of data generated from this real-time monitoring process enables for further data analysis insight than traditional measurement methods allow [4].

The device was developed with discrete component blocks, and the full diagram is presented in Figure 3a. In addition to the bio-oscillator, the sensor device implements a microcontroller based on ARM Cortex-M7 technology, configured to manage the sensor control signals, to perform the acquisition using the embedded analogue to digital converter (ADC). For fast stabilization of the oscillation process, a trigger circuit is enabled and equipped with an external communication interface via a Bluetooth module. A smart design was considered to optimize power consumption since this device is running on Li-ion batteries inside a cell culture incubator to achieve full experiment autonomy. The current battery life estimation is also provided as part of the sensor measurements.

In addition to the capabilities depicted above, a set of environmental sensors is included to also measure the relative humidity and temperature during the whole experimental process. The device employed is the Sensirion SHT21 (Amidata S. A., Pozuelo de Alarcon, Madrid, Spain), which is connected to the microcontroller via an I2C (Inter Integrated Circuit) bus as it is depicted on Figure 3a.

#### 2.1.2. Signal Acquisition and Processing

The self-sustained oscillator provides a sinusoidal wave in which oscillation parameters are directly related to the physical magnitudes under analysis. The microcontroller embeds an analog to digital converter (ADC) with 12 bits of precision, which is enough for performing a precise determination of both frequency and amplitude on the input signal. The estimation of both parameters proposed here requires signal processing based on algorithms to infer their values. It is necessary to apply discrete frequency domain analysis via fast Fourier transform (FFT) [18,19] and perform a good estimation using appropriate interpolation techniques [20]. All processing is programmed and carried out in real-time at the microcontroller.

**Figure 6:**
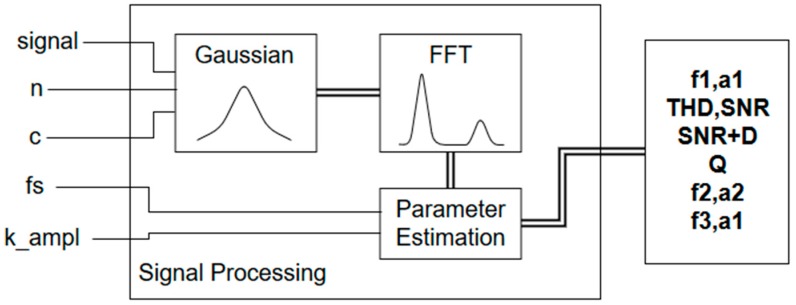
Schema for the signal processing library. The inputs for the system include the analog signal acquired from the sensor (signal), the requested fast Fourier transform (FFT) order, n, the c-parameter for the Gaussian window (c), the sampling frequency (fs) and a calibration factor (k_ampl) for the amplitude. fn and an correspond to the n-harmonic frequency and amplitude values. Total harmonic distortion (THD), signal-to-noise ratio (SNR) and signal-noise ratio+distortion (SNR+D) are also obtained. Q is a quality factor for the signal.

The signal processing implementation accounts for both time and performance optimization in terms of memory and processor resources. The architecture is depicted in Figure 6.

The system computes and estimates the signal parameters, which are related to cell culture growth (frequency and amplitude). Finally, all data gathered are sent to the gateway using the Bluetooth link.

### 2.2. Gateway

The system has been designed to minimize sensor power consumption. Three operation modes are defined in the sensor which are controlled by the gateway: standby mode, low power mode and acquisition mode.

Standby mode is used when waiting for instructions from the gateway. All devices except the microcontroller and the Bluetooth interface are turned off to minimize power consumption. The second mode is low power mode. Within this mode, the system achieves the lowest power consumption rates. Everything is off except for a peripheral on the microcontroller; a real-time clock with an alarm established by the gateway for the next measurement time. Finally, the acquisition mode must perform the measurement acquisition from the sensors and send the information back to the gateway.

**Figure 7:**
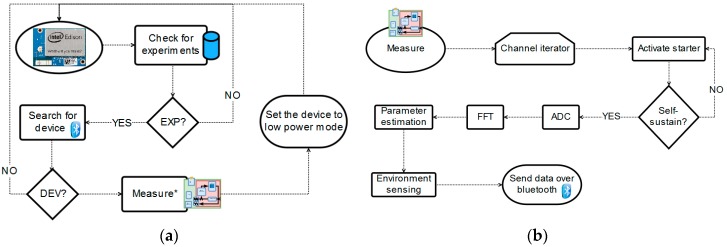
Flow diagram for (**a**) the measurement protocol (**b**) the microcontroller measurement process. EXP = experiment. DEV = device.

The trace of the system behavior is depicted on Figure 7. In Figure 7a, the flow diagram from the gateway control is depicted. The process begins with the search for a device waiting for instructions (standby mode) and a check for active experiments on the database. If all the conditions are met, then a measure is triggered (set the device to acquisition mode) and after completion, the device is set to sleep (low power mode).

Assuming a device is in Bluetooth range in standby mode and an experiment is active on the database, the process shown in Figure 7b will occur. The microcontroller iterates over the eight different channels applying the following process depicted in Figure 7b. Firstly, the start-up signal is generated on the microcontroller and applied to the oscillation loop. This improves the stabilization period for the oscillations, allowing for faster measurements in all wells. Once the oscillator is self-sustaining, the measurement is acquired using the microcontroller ADC. Finally, relative humidity and temperature sensor data are acquired (as critical factors in cell culture experiments whose stability may affect the electrode electrical response). At the end, when the device is set to low power mode, the gateway communicates with the services layer (a data server) and stores the information gathered on a database which will allow for further processing, data analysis and experiment configuration and supervision.

### 2.3. Services

The service layer for this system has been implemented bearing in mind two specific sections: a data storage service which stores all data received from the sensor in a secure database, and the user interface provided by a web application which the operator can access to configure and visualize experimental data.

#### 2.3.1. Data Server

The data server is implemented on a server established in the Higher Technical School of Computer Engineering in Seville. This server employs secure authentication to access information on a MySQL database which will store data gathered by the gateway (frequency, amplitude, temperature, humidity and battery information for each sensor device registered). Additionally, the server will store sensor device information and identification, gateway IP addresses and identifiers, experiment configuration (assigned dates, acquisition period and gateway), and user information for the web interface. 

#### 2.3.2. Web Interface

**Figure 8:**
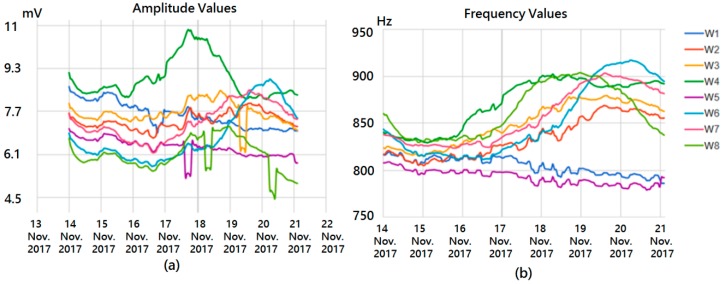
Real-time visualization on the web interface for seven days culturing N2A cells. (**a**) amplitude and (**b**) frequency values. Visualization on the website. All data are presented in real-time. The amplitude values are in millivolts and the frequency in Hertz. Each of the wells from the experiment were seeded at different density levels: empty wells containing only medium (W1, W5), 2500 cells (W2, W6), 5000 cells (W3, W7) and 10,000 cells (W4, W8).

The secure user interface is implemented on a web server via a web login interface which provides exclusive access to authorized researchers. The services offered via this interface are:

- Experiment configuration: acquisition period and dates configurable at any time. The information will be stored on the database and accessed by the gateway.
- Experiment management: start, stop, finish and delete experiments. The user can control the current experiment and finish it if necessary or stop it to change the acquisition period.
- Data visualization: real-time remote monitoring of cell culture status, as shown in Figure 8.
- Phone ready visualization: the interface was designed considering the layout of smart devices so that the researchers can easily explore, manage and follow the experiment from this kind of device.

**Figure 9:**
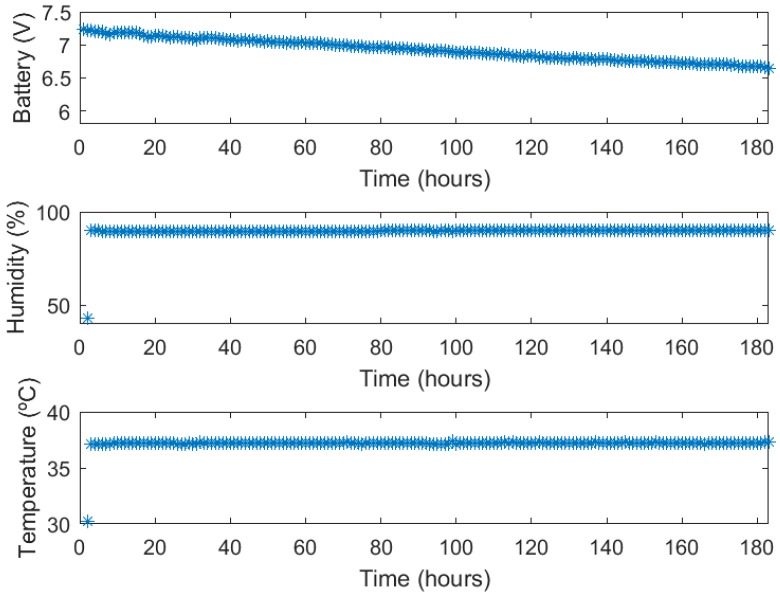
Battery voltage, relative humidity and temperature graphs.

The voltage applied to the cells is below 11 mV; this value is measured using discrete amplification for improving dynamic ranges. From Figure 8, we have selected three different points on well number six; these results will be analyzed in the following section. Environmental and battery measurements are provided in Figure 9. Relative humidity and temperature levels are stable over the whole experiment.

The batteries employed are a set of lithium ion battery packs of 5.2 Ah. According to standard discharge curves for lithium ion batteries [21], the voltage drop observed in Figure 9 corresponds to a 60% consumption in capacity (*B*_%_) which can also be expressed in mA (*B_mA_*):

$$B_{mA}=B_{\%}\cdot 5200=3120\;\mathrm{mA}$$

Finally, using the following equation, the power consumption expressed in mAh can be computed:

$$P_{mAh}=\frac{B_{mA}}{T_{hours}}=\frac{3120}{180}\approx 17\;mAh$$

## 3. Experimental Results

**Figure 10:**
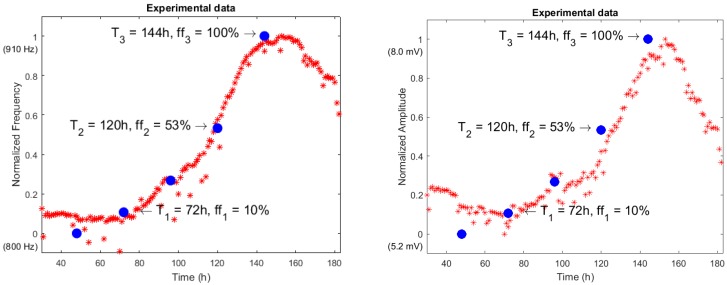
Normalized frequency (**left**) and amplitude (**right**) evolution over time (hours) for a selected well. Blue points have been obtained at set time points (T_N_) from a traditional assay using Petri plates (W6, 2500 N2A seeded cells in *t* = 0).

**Figure 11:**
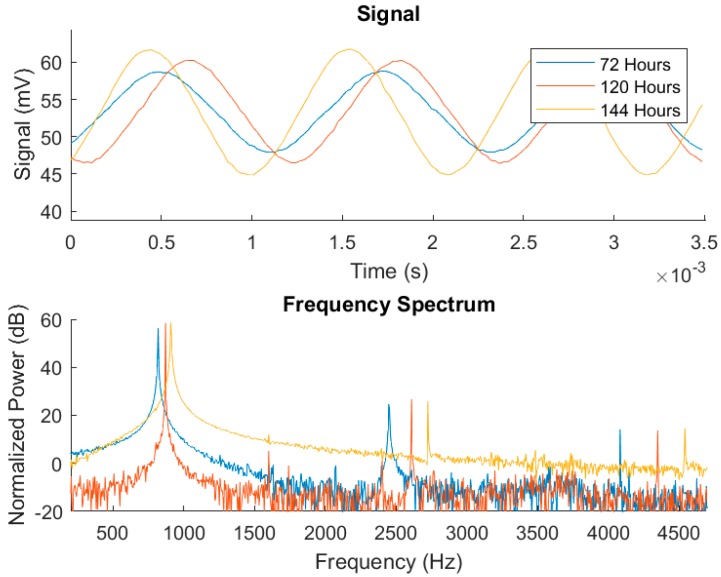
Waveforms (up) for the selected time points and the corresponding frequency spectrums (down) (W6, 2500 N2A seeded cells in *t* = 0).

From the system described above, experiments were performed using cell cultures in biomedical labs with the N2A cell line. This is a neuroblastom cell line from albino rats, employed in basic reseach on neural signaling, axon regeneration, nervous system connection repair [22], and also in chemotherapy [23]. For the purpose of illustration, a subset of the obtained output oscillation signals was selected considering different cell occupation rates. The fill factor (ff) evolution during the experiment considering sampling times of 72 h, 120 h and 144 h, corresponding to ff of 10%, 53% and 100%, is listed in Table 1. The graphic for captured points is depicted in Figure 10. For the sake of clarity, the frequency and amplitude values measured by this OBT sensor are presented normalized within the obtained experimental dynamic ranges (800–910 Hz and 5.2–8.0 mV). For the sake of validation, the points observed from the cell culture measurements with a traditional approach (employing Petri plates) in the biomedical laboratory are also presented in blue. Such time points are found on the experimental growth curves (blue points in Figure 10) by using similar cell densities in a laboratory experiment to define the characteristic growth curve for this cell line. On the other hand, the acquired waveform signals and the calculated frequency spectrums for the three cases described before are depicted in Figure 11.

## 4. Discussion

Figure 10 illustrates the normalized values for the experimental results acquired from the sensor using the N2A cell line, and a 2500/0.8 cells/cm^2^ initial density. Additionally, the blue dots represent the characteristic growth curve for this cell line, measured using a traditional approach (seeding several Petri plates and periodic cell counting) performed by the biologist team. The resulting real-time measurement suits well to the expected growth along the frequency range, proving the validity of this technique to perform cell culture monitoring growth control. Furthermore, the researchers are provided with a powerful tool including real-time visualization of the growing parameters without interrupting the experiment. Hence, the technology presented in this paper has good potential for reducing human and material costs associated with the biomedical research process on cell culture.

The experimental results presented in Figure 11 and detailed in Table 1 correspond respectively to the bio-oscillator waveform signals and frequency spectrums of the electrical response in the sample under test. The bio-oscillator signal parameters follow the growth observed on the Petri plate assay (both frequency and amplitude), proving good sensitivity for cell growth monitoring in real-time. The measured frequency better matched the predicted value compared to the amplitude, but both values provided by the sensor (depicted in Table 1) follows the predicted evolution. Also, it must be said that amplitude values (sensor amplitude in Table 1) are obtained by interfacing the cells to an operational amplifier to achieve higher dynamic range on the measurement. Other figures of merit, such as the secondary harmonics, THD or SNR, can also be used as parameters to improve measurement sensitivity.

An approached system sensitivity value (Hz/cell and mV/cell) can be estimated from the frequency and the amplitude curves in Figure 10 (Well 6). The dynamic range observed for the measured frequency and the amplitude are 79 Hz and 1.6 mV respectively, when the cell culture goes from ff = 0 to ff = 1. In this process, 0.8 cm^2^ of the well surface has been fully covered (at the confluence phase). For a circular approximated cell radius of 8 μm, the changes expected in frequency and voltage amplitude due to a cell are 0.200 mHz/cell and 4.1 nV/cell, if only a linear approach for the sensitivity calculus is considered.

The feasibility of the oscillation-based test technique has been demonstrated to sense impedance changes due to cell number increment, to validate the impedance-based system for cell culture monitoring as a result of cell attachment, and to determine the resistive properties of cell-electrode systems at low frequencies, mainly due to cell membrane capacity. The information obtained corresponds to electric current lines travelling outside the cells, since at low frequencies, cell membrane impedance is high due to its capacitive performance. Alternative optical techniques can be also employed when looking for changes in cell shape, viability, or structural composition inside cells, providing complementary information.

## 5. Conclusions

The paper describes a real-time cell culture monitoring smart sensor built upon discrete components and commercial off-the-shelf devices. This robust smart system can extract information on the current growth state from the cell culture under test through transducing cellular proliferation into electrical variables (frequency and amplitude) of a self-generated bio-oscillator.

The system architecture proposed herein is organized following the Internet of Things paradigm: devices, gateway and services as components, and is extensible to similar real-time monitoring applications. The system supports multiple sensors on a single gateway connected to the internet, and a database that serves as a data storage and control system. Three main operation modes have been designed and optimized for reduced power consumption, sampling time selection and managing multiple assays, allowing for the real-time acquisition and signal processing of sensor responses to obtain the relevant amplitude and frequency values required for the cell culture report. Experiments described with a neuroblastoma cell line (N2A) enable the proposed sensing system to be recommended as an alternative to end-point protocols, delivering comparable and useful cell culture measurement.

## 6. Patents

The work presented in this paper has been protected by a patent included on the invention registered as “Bioimpedance measurement system for wirelessly monitoring cell cultures in real time, based on an oscillation test using integrated circuits”, register number; WO2016020561A1.

## Figures and Tables

**Table 1:** Experimental values acquired from the system (W6, 2500 N2A seeded cells in *t* = 0). Frequencies, amplitudes, measured voltage, sensor amplitude, total harmonic distortion (THD) and signal-to-noise ratio (SNR).

Time (h)	Fill-Factor (ff)	Predicted Frequency (Hz) (Figure 5)	Frequency 1st Harmonic (Hz)	Predicted Amplitude (mV) (Figure 5)	Measured Cell Voltage (mV)	Sensor Amplitude (V)	THD (dBc)	SNR (dB)
72	10%	821	817	6.1	5.2	0.16	−30.0	58
120	53%	844	870	6.7	6.7	0.21	−31.5	68
144	100%	900	908	7.7	8.0	0.25	−33.5	55
